# Capybara density and climatic factors as modulators of *Ehrlichia* prevalence in questing ticks in the Iberá wetlands, Argentina

**DOI:** 10.1038/s41598-023-39557-w

**Published:** 2023-07-28

**Authors:** Ayelen T. Eberhardt, Darío E. Manzoli, Camilo Fernandez, Daniel Zurvera, Lucas D. Monje

**Affiliations:** 1 Laboratorio de Ecología de Enfermedades, Instituto de Ciencias Veterinarias del Litoral (ICIVET-Litoral), Universidad Nacional del Litoral (UNL)/Consejo Nacional de Investigaciones Científicas y Técnicas (CONICET), R.P. Kreder 2805, S3080 Esperanza, Santa Fe Argentina; 2 Facultad de Ciencias Veterinarias, Universidad Nacional del Litoral (UNL), Esperanza, Santa Fe Argentina; 3 Facultad de Bioquímica y Ciencias Biológicas, Universidad Nacional del Litoral (UNL), Santa Fe, Santa Fe Argentina

**Keywords:** Ecological epidemiology, Wetlands ecology, Parasitology

## Abstract

We evaluated the presence of *Ehrlichia* spp. in unfed capybara ticks, *Amblyomma dubitatum*, and explored its association with capybaras density, ticks density and environmental variables. We observed that in the Iberá wetlands ecoregion *A. dubitatum* is infected by "*Candidatus* Ehrlichia hydrochoerus” and in a lesser extent with an *Ehrlichia* species closely related to *Ehrlichia chaffeensis*. The frequency of "*Ca.* Ehrlichia hydrochoerus" presence in *A. dubitatum* was not associated with vector abundance, but the probability of finding "*Ca.* Ehrlichia hydrochoerus"-infected ticks increased when the density of capybaras was low two months before. We hypothesize that when the density of capybaras decreases, *A. dubitatum* immature stages may seek out alternative hosts one of which could exhibit high realized reservoir competence for "*Ca.* Ehrlichia hydrochoerus", leading to an increased prevalence of this ehrlichiae in questing *A. dubitatum*. High minimum temperatures and high cumulative rainfall in the time period previous to tick collection (15 to 60 days) were positively correlated with the prevalence of "*Ca.* Ehrlichia hydrochoerus" infection in *A. dubitatum*. Our results suggest that a combination of factors (both biological and abiotic) could raise the risk of human exposure to tick-borne *Ehrlichia* in the Iberá wetlands ecoregion.

## Introduction

Emerging infectious diseases pose a significant burden on the global economy and public health^1^. The majority of these emerging infectious diseases are zoonoses originating from wild animals^2^. Among arthropod vectors, ticks are responsible for transmitting the greatest diversity of pathogens that affect humans, livestock and companion animals^3^. In the northern hemisphere, Ixodidae transmits the pathogens that cause Lyme disease (*Borrelia burgdorferi*), human monocytic ehrlichiosis (*Ehrlichia chaffeensis*) and human babesiosis (*Babesia microti*). None of these tick-borne parasites is transmitted transovarially; hence, larvae or nymphs must acquire the infection during a blood meal on a infected host^4–6^. The probability that a particular host species transmit infection to a feeding vector is often called realized reservoir competence^7^, and it varies between different host species^8^. Since most pathogens can infect multiple hosts, the relative abundance of competent and incompetent host species in a community can cause the abundance of pathogens to vary^9^. The abundance of infected ticks actively seeking a host is affected by both the abundance of ticks in the vegetation and the prevalence of the evaluated pathogen. These factors exhibit significant temporal fluctuations attributed to variations in climatic conditions and changes in the abundance of vertebrate hosts^10,11^. Understanding the complex mechanisms underlying these fluctuations is crucial, as climatic conditions, vertebrate hosts, ticks, and tick-borne microorganisms form intricate biological networks with multiple interactions^12^. Investigating these interdependencies would enhance our comprehension of the fluctuations in the distribution and incidence of tick-borne parasites.

*Ehrlichia* spp. are intracellular Gram-negative bacteria of medical and veterinary importance that infect monocytes, neutrophils, or endothelial cells, depending on the species involved^4^. The genus *Ehrlichia* comprises six formally recognized tick-transmitted species: *Ehrlichia canis*, *Ehrlichia muris*, *E. chaffeensis*, *Ehrlichia ewingii*, *Ehrlichia minasensis*, and *Ehrlichia ruminantium*. Additionally, several different strains of putative novel *Ehrlichia* species have been molecularly detected recently, although their taxonomic positions are still not clearly defined^13–18^.

The tick *Amblyomma dubitatum* Neumann is distributed in the biogeographic provinces of Chaco, Pampa, Parana Forest and Atlantic Forest in the Neotropical region of southern South America^19,20^. All stages of *A. dubitatum* feed predominantly on capybaras (*Hydrochoerus hydrochaeris* L.)^19,21^. Moreover, immature stages can use small vertebrates as alternative hosts^22^, while all *A. dubitatum* stages were recorded biting humans^19,23^. In the Iberá wetlands ecoregion (Corrientes province, northeastern Argentina), capybaras are present at high densities, living in close proximity to farms and urban settlements^24,25^. This results in an extensive human-domestic-wildlife interface that may pose a potential risk to public health and animal husbandry.

The aim of this study was to evaluate the infection rate of *Ehrlichia* species in unfed *Amblyomma dubitatum* ticks collected from vegetation and investigate its association with capybara density, tick density, and environmental parameters in protected areas within the Iberá wetlands ecoregion in Argentina.

## Results

**Figure 1:**
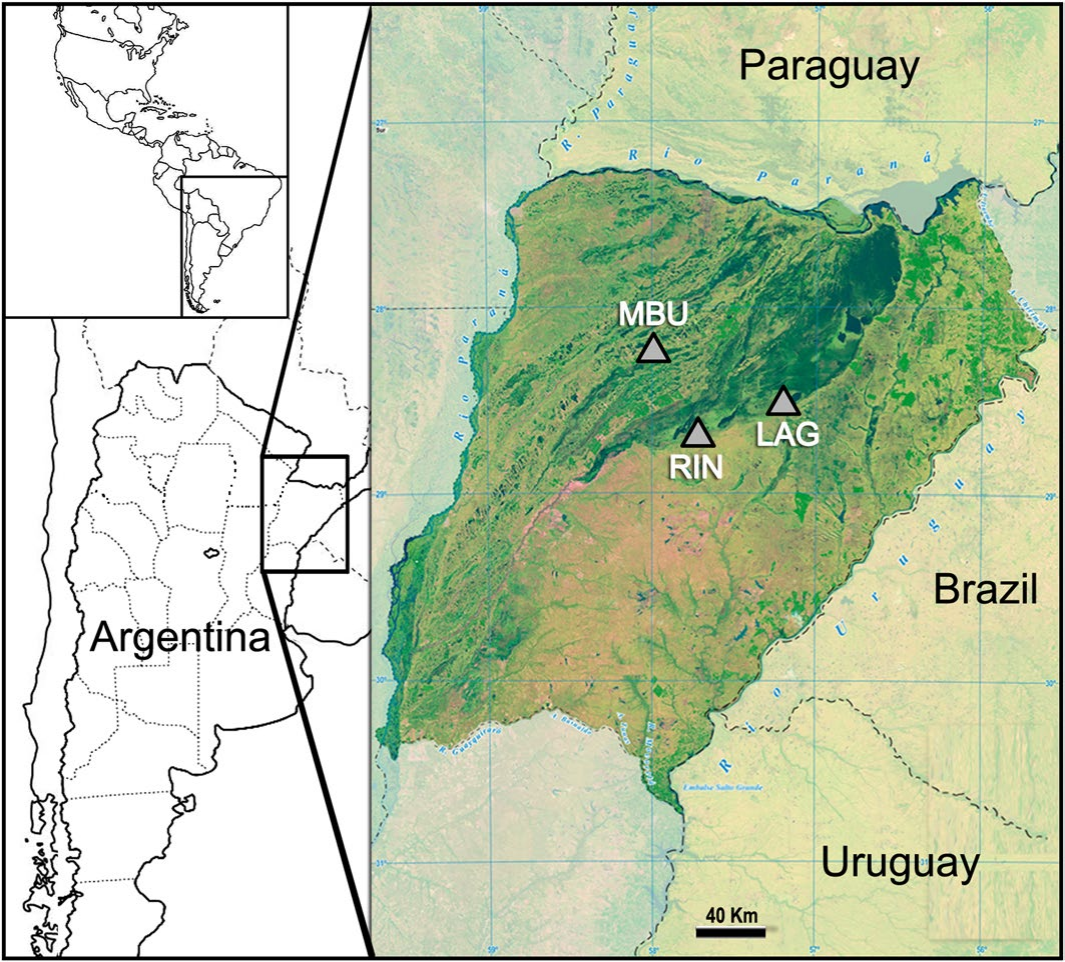
Map of part of South America showing the sampling sites in Iberá wetlands ecoregion, Argentina. MBU, National Park Mburucuyá; RIN, Rincón del Socorro; LAG, Laguna Iberá. Both sites RIN and LAG are part of National Park Iberá. The map was generated with the software GIMP version 2.10.34 (https://www.gimp.org/) based on images from the Instituto Geográfico Nacional de la República Argentina (https://www.ign.gob.ar/) licensed under the Creative Commons Public Domain license (https://creativecommons.org/licenses/by-sa/4.0/).

During the twelve sampling sessions, a total of 13,941 ticks were collected from the protected areas studied (Fig. 1). These ticks were identified as *A. dubitatum*, *Rhipicephalus microplus* Canestrini, *Amblyomma triste* Koch, *Haemaphysalis juxtakochi* Cooley and *Amblyomma tigrinum* Koch. The total number of tick specimens analyzed individually and in pooled samples, discriminated by species and stage is presented in Table 1.

**Table 1:** Number of tick specimens analyzed individually and in pooled samples, discriminated by species and stage.

Stage	Analyzed	*Amblyomma dubitatum*	*Amblyomma tigrinum*	*Amblyomma triste*	*Haemaphysalis juxtakochi*	*Rhipicephalus microplus*
Larvae	Individually	34	0	4	1	4
	Pooled*	449/11,180 (2–250)	0	9/113 (2–20)	0	18/195 (2–18)
	Total^1^	11,214 (82.43%)	0 (0%)	117 (90.7%)	1 (20%)	199 (98.5%)
Nymphs	Individually	1246	1	10	4	0
	Pooled*	310/1034 (2–6)	0	0	0	1/3 (3)
	Total^1^	2280 (16.76%)	1 (100%)	10 (7.75%)	4 (80%)	3 (1.5%)
Adults	Individually	110	0	2	0	0
	Pooled*	0	0	0	0	0
	Total^1^	110 (8.08%)	0	2 (1.55)	0	0
Total of ticks collected by species		13,604	1	229	5	202

*Number of pools/specimens analyzed. The range of specimens per pool is indicated in parentheses.

^1^Total number of each species stage ticks. The percentage is indicated in parentheses.

During the two–year period, the seasonal distribution of questing *A. dubitatum* exhibited a similar pattern for all stages. Questing larvae and nymphs were collected throughout the entire year. The peak of questing larvae of *A. dubitatum* occurred in autumn, while nymphs showed peaks in winter and spring. Adult *A. dubitatum* reached its peak during summer see^26^. Fourteen *A. dubitatum* samples (4 adults, 8 nymphs and 2 nymph pools) resulted positive for Anaplasmataceae 16SrRNA PCR presenting melting curve peaks matching those observed for *Ehrlichia* sp. (86.2 °C) see ^26^. No amplification was observed in samples from *A. triste*, *H. juxtakochi, R. microplus* and *A. tigrinum* samples.

**Figure 2:**
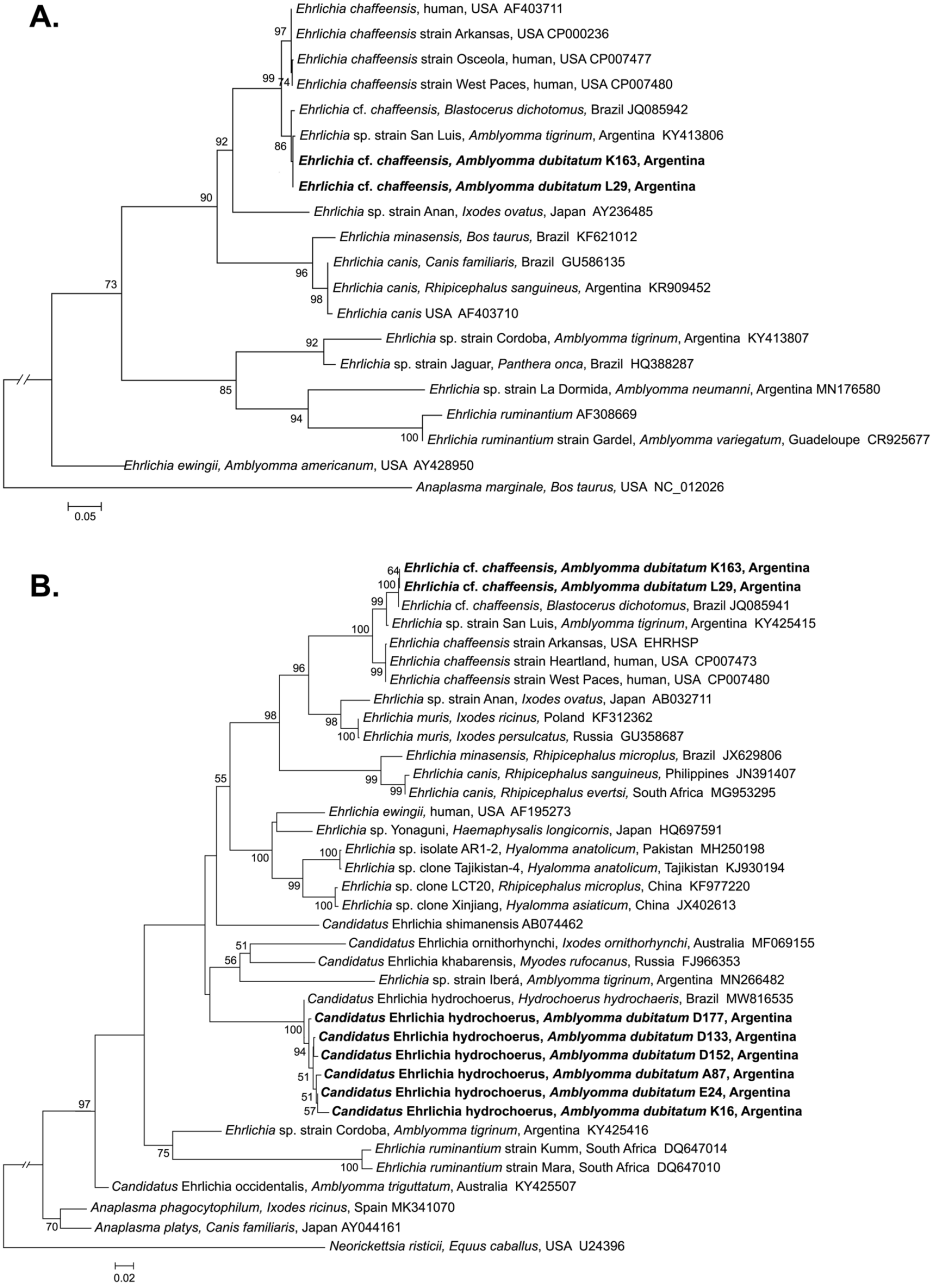
Maximum-likelihood trees constructed from (**A**) *dsb* and (**B**) *groEL* sequences of *Ehrlichia* species infecting *Amblyomma dubitatum* in Argentina compared with reference strains. Best-fitting substitution models using the Maximum-Likelihood model test were determined with the Akaike Information Criterion. Tamura 3-parameter with a discrete Gamma distribution and with evolutionarily invariable sites was selected as the best model for *dsb* and Hasegawa-Kishino-Yano with a discrete Gamma distribution was selected as the best model for *groEL*. Numbers represent bootstrap support generated from 1000 replications. GenBank accession numbers are shown. Boldface indicates the strain identified in this study. Scale bars indicate nucleotide substitutions/site.

Two of the 16SrRNA-positive *A. dubitatum* samples (1 nymph, 1 nymph pool) amplified both the *dsb* and *groEL* targets. The 374-bp fragment of the *dsb* gene obtained from these two samples showed 99.7% identity to the corresponding sequence of *Ehrlichia* sp. strain San Luis (MH261375) and 97.6% identity to the corresponding sequence of *Ehrlichia chaffeensis* str. West Paces (CP007480). The 1196-bp fragment of the *groEL* gene obtained showed 99.9% identity to *Ehrlichia* cf. *chaffeensis* from marsh deer (JQ085941) and 98.1% identity to the corresponding sequence of *Ehrlichia chaffeensis* str. West Paces (CP007480). Phylogenetic analyses using the *dsb* and *groEL* sequences from these two samples placed the *Ehrlichia* sp., hereinafter called *Ehrlichia* cf. *chaffeensis* from *A. dubitatum*, in the same clade as *Ehrlichia* sp. strain San Luis previously reported to infect *A. tigrinum*^13^ and *Amblyomma parvum*^27^, as well as *Ehrlichia* cf. *chaffeensis* reported in free-ranging marsh deer in Brazil (Fig. 2A,B). This South American clade of *Ehrlichia* cf. *chaffeensis* strains was strongly supported as the sister clade to a clade consisting of several *Ehrlichia chaffeensis* strains reported in North America (Fig. 2A,B).

The remaining 16SrRNA-positive samples amplified only the *groEL* gene. The obtained sequences (952–1109 bp) showed 99.22% to 99.40% identity to "*Candidatus* Ehrlichia hydrochoerus" (MW816535), which was previously reported to infect capybaras in Brazil^18^. Phylogenetic analysis using these *groEL* sequences placed the detected ehrlichial agent, hereinafter referred to as "*Ca.* Ehrlichia hydrochoerus" from *A. dubitatum*, in the same clade as the novel "*Ca.* Ehrlichia hydrochoerus" (Fig. 2B) and close to *Ehrlichia* sp. strain Iberá reported to infect *A. tigrinum* in the same region^14^.

**Figure 3:**
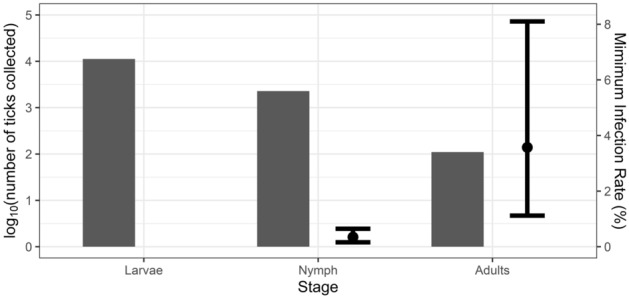
Number of *Amblyomma dubitatum* ticks collected (log_10_ scale) and Minimum Infection Rate of "*Candidatus* Ehrlichia hydrochoerus" from *A. dubitatum* for each life stage. The point depicts the estimated prevalence and the error bars show the 95% confidence interval.

The frequency of infection with "*Ca.* Ehrlichia hydrochoerus" in *A. dubitatum* varied depending on the life stage of the tick. Lower infection rates were observed in nymphs (MIR: 0.35%, CI 95% 0.16–0.65) compared to adult ticks (MIR: 3.57%; CI 95% 1.12–8.1). The MIR of "*Ca.* Ehrlichia hydrochoerus" in adults was 10.6 times higher than in nymphs (*p-value* < 0.001) (Fig. 3). *Ehrlichia* cf. *chaffeensis* from *A. dubitatum* was only detected in nymphs (MIR: 0.014%, CI 95% 0.002–0.044).

Statistical analyses were conducted to explore associations between the presence of *Ehrlichia* in *A. dubitatum* and host, vector and abiotic variables. The analysis utilized data from both nymphs and adults combined, as there is no evidence of transovarial transmission of *Ehrlichia* (unfed questing larvae cannot be infected). It is important to note that the number of ticks infected with *Ehrlichia* cf. *chaffeensis* from *A. dubitatum* was insufficient for robust comparisons. Therefore, the statistical analyses were focused solely on ticks infected with "*Ca.* Ehrlichia hydrochoerus".

**Figure 4:**
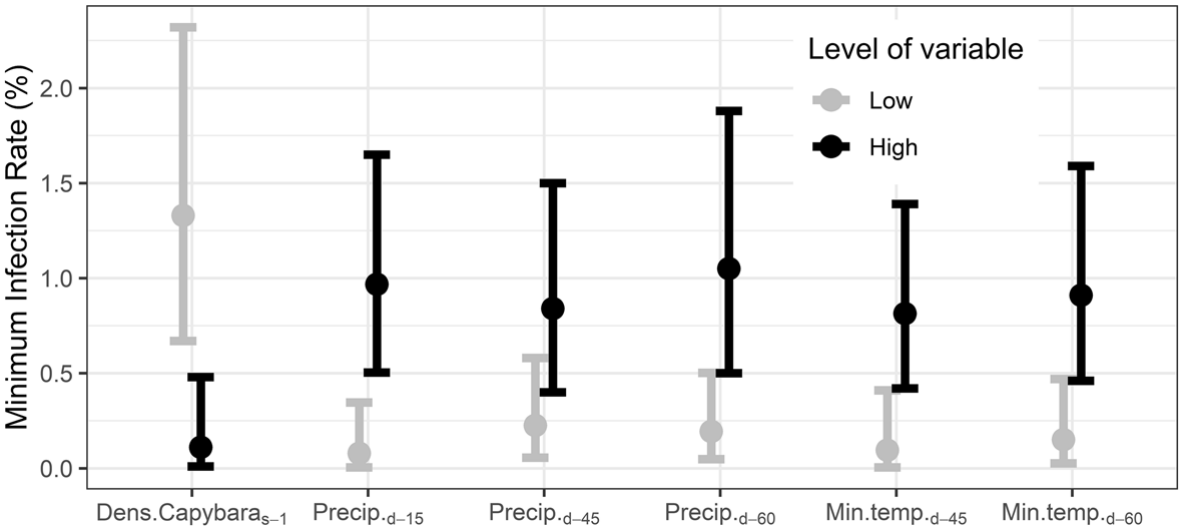
Minimum Infection Rate (MIR) of “*Candidatus* Ehrlichia hydrochoerus” in *Amblyomma dubitatum* ticks related to low and high levels of climatic variables (at different lag times) and capybara density in S_-1_. The point depicts the estimated median value of MIR and the error bars show the 95% confidence interval.

The density of capybaras in the previous session (S_-1_) had a negative effect on the frequency of "*Ca.* Ehrlichia hydrochoerus" infection in ticks (Fig. 4). In the low-capybara density group at S_-1_ (1.55 to 10.5 capybaras per linear km at S_-1_), the MIR for "*Ca.* Ehrlichia hydrochoerus" was 1.33% [95% CI 0.67–2.32]. In contrast, in the high-capybara density group at S_-1_ (between 12.4 and 55.0 animals per linear km at S_-1_), the MIR for "*Ca.* Ehrlichia hydrochoerus" was 0.11% [CI 95% 0.01–0.48]. This indicates a 12.2-fold change in the prevalence of "*Ca.* Ehrlichia hydrochoerus"-infected ticks collected in sites where the density of capybaras at S_-1_ was low (*p-value* = 0.0173) (Fig. 4).

Regarding climate variables, the accumulated rainfall in the last 15, 45 and 60 days prior to each sampling (with median values of 27, 63 and 101 mm, respectively) and the average minimum temperature at 45 and 60 days prior to each sampling (with median values of 15.8 and 15.2 °C, respectively) all showed positive effects on the frequency of "*Ca.* Ehrlichia hydrochoerus" presence in *A. dubitatum* (Fig. 4). The frequency of "*Ca.* Ehrlichia hydrochoerus" presence in *A. dubitatum* was significantly higher for high values compared to low values of each variable: accumulated precipitation (d_-15_: 12.43-fold, *p-value* = 0.016; d_-45_: 3.76-fold, *p-value* = 0.047; d_-60_: 5.43-fold, *p-value* = 0.011) and average minimum temperature (d_-45_: 8.65-fold, *p-value* = 0.039; d_-60_: 5.6-fold, *p-value* = 0.020). In other words, elevated minimum temperatures and increased accumulative rainfall in the specified time periods were positively associated with the prevalence of "*Ca.* Ehrlichia hydrochoerus" presence in *A. dubitatum*. No significant associations were observed between "*Ca.* Ehrlichia hydrochoerus" presence in *A. dubitatum* and other climatic variables, season, larvae and nymphs abundance.

## Discussion

Our findings demonstrate that *A. dubitatum* in the Iberá wetlands ecoregion is infected by two genetically distinct *Ehrlichia* species. One of these species is closely related to the zoonotic pathogen *Ehrlichia chaffeensis*^28^, while the other is similar to the novel "*Ca.* Ehrlichia hydrochoerus"^18^. Phylogenetic analysis using both the *dsb* and *groEL* loci placed *Ehrlichia* cf. *chaffeensis* from *A. dubitatum* in the same clade as *Ehrlichia* sp. strain San Luis and *Ehrlichia* cf. *chaffeensis* from marsh deer, and in proximity to several *E. chaffeensis* strains from the USA. Further research utilizing additional phylogenetic markers is necessary to determine whether all of these South American ehrlichiae, closely related to *E. chaffeensis*, are indeed distinct species or constitute an *Ehrlichia chaffeensis* sensu lato complex, as has been observed for other tick-transmitted rickettsial pathogens in the region^29^.

Using *groEL* sequences, the other detected ehrlichial agent was identified as "*Ca.* Ehrlichia hydrochoerus". However, in our study, we were unable to amplify the *dsb* gene of "*Ca.* Ehrlichia hydrochoerus" from *A. dubitatum*. This is consistent with the hypothesis that the commonly targeted *dsb* sequence is highly polymorphic and may not be amplifiable in certain ehrlichiae^14^. Coincidentally, similar challenges were encountered by Vieira et al*.*^18^ and Eberhardt et al*.*^14^ when attempting to amplify *dsb* sequences from "*Ca.* Ehrlichia hydrochoerus" infecting capybaras and the phylogenetically related *Ehrlichia* sp. strain Iberá infecting *A. tigrinum*, respectively. In a recent study, we reported the detection of another member of the Anaplasmataceae family closely related to *Anaplasma odocoilei* in *A. dubitatum*^26^. To fully understand the potential role of *A. dubitatum* as a vector of these Anaplasmataceae, further experiments are needed to determine its vector competence. This is particularly important given the zoonotic potential of *Ehrlichia* cf. *chaffeensis* and the fact that *A. dubitatum* is known to parasitize humans in all of its developmental stages^19,23,30^.

The capybara is the principal host of all stages of *A. dubitatum*^19^. In Brazil, two studies were conducted to investigate the presence of ehrlichial agents in capybaras and their associated ticks. In central-western Brazil, Neves et al*.*^31^ reported the absence of ehrlichiae in capybara blood and their associated *A. dubitatum*. In southern Brazil, Vieira et al*.*^18^ found that "*Ca.* Ehrlichia hydrochoerus" infects capybara blood but not the salivary glands of *A. dubitatum* feeding on these capybaras. However, it is important to note that both studies analyzed a small number of *A. dubitatum* (132 and 11 samples, respectively). In contrast, our study analyzed a large number of ticks from different populations over time, which enabled us to identify the presence of two distinct ehrlichial agents infecting host-seeking *A. dubitatum*.

Despite capybaras from the same populations in this study being highly infested by *A. dubitatum*^32^ and infected by "*Ca.* Ehrlichia hydrochoerus"^33^, the prevalence of the ehrlichial agent in the ticks collected from the same site was found to be very low (0.59% of the 2390 *A. dubitatum* nymphs and adults). Considering the possibility of *A. dubitatum* having a low susceptibility to "*Ca.* Ehrlichia hydrochoerus" infection, it is plausible that the bacterial loads present in the capybara's blood might not be sufficient to facilitate infection in the tick. In this scenario, capybaras may play a dual role by acting as key reproductive hosts for *A. dubitatum* and potentially as incompetent or low realized competent hosts for "*Ca.* Ehrlichia hydrochoerus". Further research is necessary to test this hypothesis and better understand the specific dynamics between capybaras, *A. dubitatum*, and "*Ca*. Ehrlichia hydrochoerus".

Our study revealed that the frequency of "*Ca.* Ehrlichia hydrochoerus" presence in *A. dubitatum* was not dependent on the density of the tick vector. However, we observed that the likelihood of finding "*Ca.* Ehrlichia hydrochoerus"-infected *A. dubitatum* was higher in sites with previously low capybara densities. Since ehrlichiae are not transmitted transovarially^4^, unfed nymphs and adults of *A. dubitatum* can acquire the infection only through transstadial transmission, which occurs when larvae or nymphs become infected by feeding on a competent reservoir host. Despite *A*. *dubitatum* having a one-year life cycle, multiple cohorts can coexist within the same population in the Iberá wetlands ecoregion^26,34^. The pre-moult period for larvae and nymphs of *A. dubitatum* ranges from 23 to 60 days and 25 to 50 days, respectively, throughout most of the year^34^. Considering that the feeding period for *A. dubitatum* larvae and nymphs is approximately 6 days^35^, it can be inferred that the majority of larvae and nymphs found in the vegetation during one sampling session will have undergone moulting into nymphs or adults, respectively, two months later (which corresponds to our inter-sampling session interval). Our findings suggest that when the density of capybaras (the primary host of all stages of *A. dubitatum*) was low in a particular site during the first sampling session (S_-1_), the prevalence of "*Ca.* Ehrlichia hydrochoerus"-infected *A. dubitatum* in the same site two months later (S) was higher compared to sites where the density of capybaras was high during the initial sampling session (S_-1_). The immature stages of *A. dubitatum* in the Iberá wetlands have been reported to feed on various vertebrate hosts in addition to capybaras^22^. Common alternative hosts include the caviine *Cavia aperea*, the sigmodontines *Akodon azarae* and *Oligoryzomys flavescens*, and the marsupial *Monodelphis dimidiata*^22^. Interestingly, these alternative hosts tend to have smaller home ranges compared to the area where capybara density was estimated^36–38^. Based on this observation, we propose a hypothesis that when the density of capybaras decreases in a particular site, the immature stages of *A. dubitatum* may seek out alternative hosts for blood meals. Consequently, larvae and nymphs of *A. dubitatum* that feed on these alternative hosts would undergo moulting within the same site, leading to the presence of its nymphs and adults stages questing for hosts in the same site as well. A study on the parasitic *Philornis* botfly, another host-seeking arthropod parasite, and its multiple bird hosts, demonstrated that the parasite selects alternative hosts only when the principal host is insufficiently available^39^. In the context of our study, it is possible that at least one of the alternative hosts parasitized by immature stages of *A. dubitatum* exhibits high realized reservoir competence for "*Ca.* Ehrlichia hydrochoerus", leading to an increased prevalence of this ehrlichiae in questing *A. dubitatum* nymphs and adults two months later. For *Ehrlichia* cf*. chaffeensis*, the low prevalence observed in *A. dubitatum* prevented any statistical analysis. Nevertheless, considering that capybaras have not been previously reported to be infected by this pathogen, it is plausible that *A. dubitatum* also acquires *Ehrlichia* cf. *chaffeensis* by feeding on alternative hosts. The marsh deer is the only vertebrate in the region known to be infected by *Ehrlichia* cf. *chaffeensis*^40,41^. However, it should be noted that the marsh deer is not parasitized by any of the stages of *A. dubitatum*^19,21^. Co-feeding transmission of *Ehrlichia muris–*like agent was demonstrated for *Ixodes scapularis* larvae feeding along with infected-nymphs^42^. Thus, a tick previously fed on an infected marsh deer could transmit *Ehrlichia* cf. *chaffeensis* by co-feeding to *A. dubitatum* in the same capybara host. In the Iberá wetlands ecoregion, *A. triste* is the only tick species reported to parasitize both marsh deer and capybaras^22,32^. However, *A. triste* parasitism of capybaras is infrequent^32^ and this tick species solely feeds on marsh deer during the adult stage^19,21^. In addition, it is important to consider that other large mammals (such as *Sus scrofa*, *Axis axis*, *Lepus europaeus, Myrmecophaga tridactyla*) as well as birds are also utilized as alternative hosts by the immature stages of *A. dubitatum*, albeit to a lesser extent^19,21,22^. Therefore, the possibility that these hosts may serve as competent reservoir for "*Ca.* Ehrlichia hydrochoerus" and/or *Ehrlichia* cf*. chaffeensis* should not be disregarded.

Regarding environmental variables, we showed a positive correlation between high minimum temperatures and previously cumulated rainfall with the prevalence of "*Ca.* Ehrlichia hydrochoerus" in *A. dubitatum*. Abiotic factors not only have direct effects on tick fitness but may also modulate pathogen development and growth in ticks^43–45^. Parasites are not harmless to their hosts, and this holds true for tick-borne rickettsial pathogens as well, which can cause a decrease in both tick survival^46^ and moulting success^47^. Given that the prevalence of "*Ca.* Ehrlichia hydrochoerus" does not depend on *A. dubitatum* density, it can be hypothesized that the observed positive relationship may be attributed to the increased survival of "*Ca.* Ehrlichia hydrochoerus"-infected *A. dubitatum* under favorable conditions. Possible explanations for these observations include heightened tolerance of the infection in hosts that are in good condition^48^ or temperature-driven changes in the transcriptional profile of the bacteria that affect virulence^49^. To test these hypotheses, additional field and laboratory experiments are required.

In summary, our findings suggest that a combination of biological factors such as capybara density, along with abiotic factors including temperature and accumulated precipitation, may contribute to an elevated risk of human exposure to tick-borne *Ehrlichia* in the Iberá wetlands ecoregion.

## Methods

### Study area

The study was conducted in the Iberá wetlands ecoregion, which encompasses a system of estuaries, baths, shallow lakes, and watercourses interconnected within an area of approximately 40.415 km^2^. The primary source of water in the wetlands is rainfall, with an average historical precipitation of 1700–1800 mm. During the summer, rainfall is slightly higher (600–700 mm) compared to other seasons. The climate in the region is humid and subtropical. The monthly average minimum temperature in June and July is around 16 °C and 17 °C, respectively, while the average maximum temperature occurs in January and February, ranging between 27 °C and 28 °C.

The questing ticks used in this study were collected from two protected areas known to have large populations of capybaras^25^. The collection sites were the National Park Mburucuyá (site MBU: 28°03′ S, 58°06′W) and the National Park Iberá (sites Rincón del Socorro, RIN: 28°39′S, 57°26′W and Laguna Iberá, LAG: 28°33′S, 57°13′W), as shown in Fig. 1.

### Data collection

Ticks were collected for this study every two months over a period of two years, from June 2015 to June 2017. Two methods, carbon dioxide (CO_2_) traps and dragging, were used for tick collection. For the CO_2_ trap method, each trap consisted of a 1 m^2^ white flannel with a perforated Styrofoam pot placed at the center, containing approximately 500 g of pelletized dry ice as CO_2_ source. At each sampling site, a body of water was chosen, and a 100 m transect was established along the coastline of that body. Ten CO_2_ traps were placed along this transect at intervals of 10 m. The traps remained active for 90 min, and were checked for ticks every 10 min during this period. In addition, drag sampling was conducted in three parallel 100 m transects, located outside the transect where the CO_2_ traps were placed. During dragging, a 1 × 1.5 m cloth was dragged along the ground, and ticks were collected by inspecting the cloth approximately every 5 m. The tick collection surveys were carried out during the mid-morning and mid-afternoon periods, specifically avoiding the hottest hours of midday.

The taxonomic determination of larvae, nymphs and adult ticks collected was conducted following Joan^50^, Guglielmone and Viñabal^51^ and Nava et al*.*^19^, and by comparison with known laboratory-reared specimens deposited in the tick collection of INTA Rafaela, Argentina. Then, all ticks were processed for DNA extraction by a boiling technique^52^. Adult ticks were processed individually, while nymphs and larvae were processed either individually or in pools. The pooling of nymphs and larvae was based on date, trap, species and abundance.

All samples were screened for *Ehrlichia* infection using a previously described real-time PCR assay that targets the 16SrRNA gene^27^. This assay is capable of identifying the genus involved through melting curve analysis^26^.

Positive samples were further tested by amplifying the *dsb* and *groEL* genes, as described^53,54^. The integrity of DNA obtained from ticks was checked using primers that amplify a portion of the arthropod 16SrRNA^55^, as previously described^52^. For all PCR reactions, positive controls (*Ehrlichia canis* or *Amblyomma triste*) and negative controls (molecular-grade water) were included. The resulting PCR products were sequenced directly using amplifying primers. Phylogenetic analyses were conducted using the Maximum-likelihood (ML) method with MEGA 7.0.

To estimate the relative density of capybaras at each site (number of capybaras per kilometer of shoreline), we utilized the frequency of fresh capybara faeces (pellet groups) observed along the shoreline. This indirect method, known as pellet group count, is a reliable indicator of capybara presence^56^. Alongside tick sampling, transects parallel to the shoreline were examined for the presence of fresh capybara pellet groups. For National Park Mburucuyá, two 300-m-long transects were used, while for Laguna Iberá and Rincón del Socorro, three 100-m-long transects were used. The transects were 5 m wide and separated by 200 m from each other. Transects were established based on the terrain limitations such as the shape of the water body and the presence of dense vegetation. The estimation of capybara density from pellet group counts was done using the modified Eberhard and Van Etten^57^ model. The capybara defecation rate used in the calculation was 4.4 faeces per individual per day, which was estimated from a previous study conducted in the same area^56^.

To estimate the abundance of *A. dubitatum* present in the study areas, the total number of ticks collected was counted and categorized by stage, season and site. The climatic variables recorded were cumulative precipitation and average minimum, mean and maximum temperature (meteorological records were obtained from: https://centrales.bolsacer.org.ar/).

### Statistical analysis

The response variable was the presence or absence of *Ehrlichia* DNA in ticks, a dichotomous variable. The independent variables used included larvae and nymph abundance, capybara density, site, season (southern hemisphere), precipitation levels and temperature. All variables were transformed into dichotomous variables by dividing the values into high and low categories based on the median value. Variables that incorporated time delays (time lags) were analyzed by considering the number of previous samplings (indicated as S) or days (indicated as d), depending on the specific variable being analyzed. The variables “larvae abundance” and “nymph abundance” were evaluated at a time lag S_-1_, while “capybara density” was evaluated at time lags S_-1_ and S_-2_, representing approximately 60 and 120 days, respectively. The variables “temperature” and “precipitation level” were evaluated using time lags of d_-15_, d_-30_, d_-45_ and d_-60_. Additionally, the time lag ranges d_-15,-30_; d_-30,-45_ and d_-45,-60_ were considered. For the statistical analysis, all DNA tick samples were treated as pools, while individually processed ticks were considered as a pool with size equal to 1.

The frequency of *Ehrlichia* presence in ticks was expressed as the Minimum Infection Rate (MIR), defined as the lower limit of the true infection rate. The MIR was calculated as the ratio of the number of positive pools to the total number of ticks tested. The MIR assumes that only one infected individual exists in a positive pool^58^. The presence of *Erhlichia* in pool ticks samples was adjusted for the size of each pool and analyzed using logistic regression models. All statistical analyses were performed using the PoolTestR package^59^ in R version 4.2.1^60^.

## Data Availability

The sequences generated and analysed during the current study are available in the GenBank repository, accession numbers OR001765-OR001775.
